# Rubinstein–Taybi Syndrome in a Filipino Infant with a Novel *CREBBP* Gene Pathogenic Variant

**DOI:** 10.1155/2022/3388879

**Published:** 2022-05-21

**Authors:** Rhea Camille R. Yumul, Mary Anne D. Chiong

**Affiliations:** Department of Pediatrics, University of Santo Tomas Hospital, España Boulevard, Manila, Philippines

## Abstract

Rubinstein–Taybi syndrome (RSTS) is a rare genetic disorder characterized by dysmorphic facial features, broad thumbs and halluces, intellectual disability, and postnatal growth retardation. This report presents a male infant with microcephaly and characteristic facial features, namely, low anterior hairline, hirsutism, thin upper lip and micrognathia, broad thumbs and first toes, cryptorchidism, recurrent pneumonia, developmental delay, and growth retardation. Genetic testing showed a novel pathogenic variant in the *CREBBP* gene which is consistent with the clinical diagnosis of RSTS.

## 1. Introduction

Rubinstein–Taybi syndrome is a rare multiple congenital anomaly syndrome characterized by dysmorphic facial features such as downslanted palpebral fissures, low-hanging columella and broad nasal bridge, broad thumbs and halluces, intellectual disability, and postnatal growth retardation [1, 2]. It also presents with cardiac and genitourinary abnormalities, recurrent infections, feeding difficulties, constipation, and hearing loss [2, 3]. The name of the syndrome comes from two physicians, Jack Rubinstein, a pediatrician, and Hooshang Taybi, a radiologist, who initially independently identified cases presenting with the aforementioned features and eventually published their findings together in 1963. The name Rubinstein–Taybi syndrome was suggested to be used in 1964 and was eventually adopted in subsequent reports to date [3].

The estimated birth prevalence is 1 in 125,000 live births [3], and in the Philippines, based on the registry of the Institute of Human Genetics of the University of the Philippines-National Institute of Health, there were 13 clinically diagnosed cases recorded from the year 2000 to 2020. It occurs sporadically in most cases and is associated with loss-of-function mutations in the homologous genes cAMP-response element binding protein (CREB)-binding protein (*CREBBP*) and EA1 binding protein p300 (*EP300*) in 50–75% of cases [1, 2]. In sporadic cases, the recurrence risk for a couple with a previous child with RSTS is 0.1% [2]. We report a Filipino male infant diagnosed with RSTS through his characteristic physical features and confirmed by gene-targeted sequencing.

## 2. Case Report

A newborn male was born full term at 38 weeks to a 22-year-old primigravid mother via normal spontaneous delivery. The mother had a history of heavy alcohol intake in the first 3 months of pregnancy and active cigarette smoking in the first 6 months of pregnancy. The rest of the prenatal course was uncomplicated, with regular prenatal check-ups. The antenatal ultrasonography and congenital anomaly scan done were unremarkable. He was the first child of a healthy nonconsanguineous couple of Filipino descent. Both parents had no physical abnormalities, and the family history was noncontributory.

**Figure 1:**
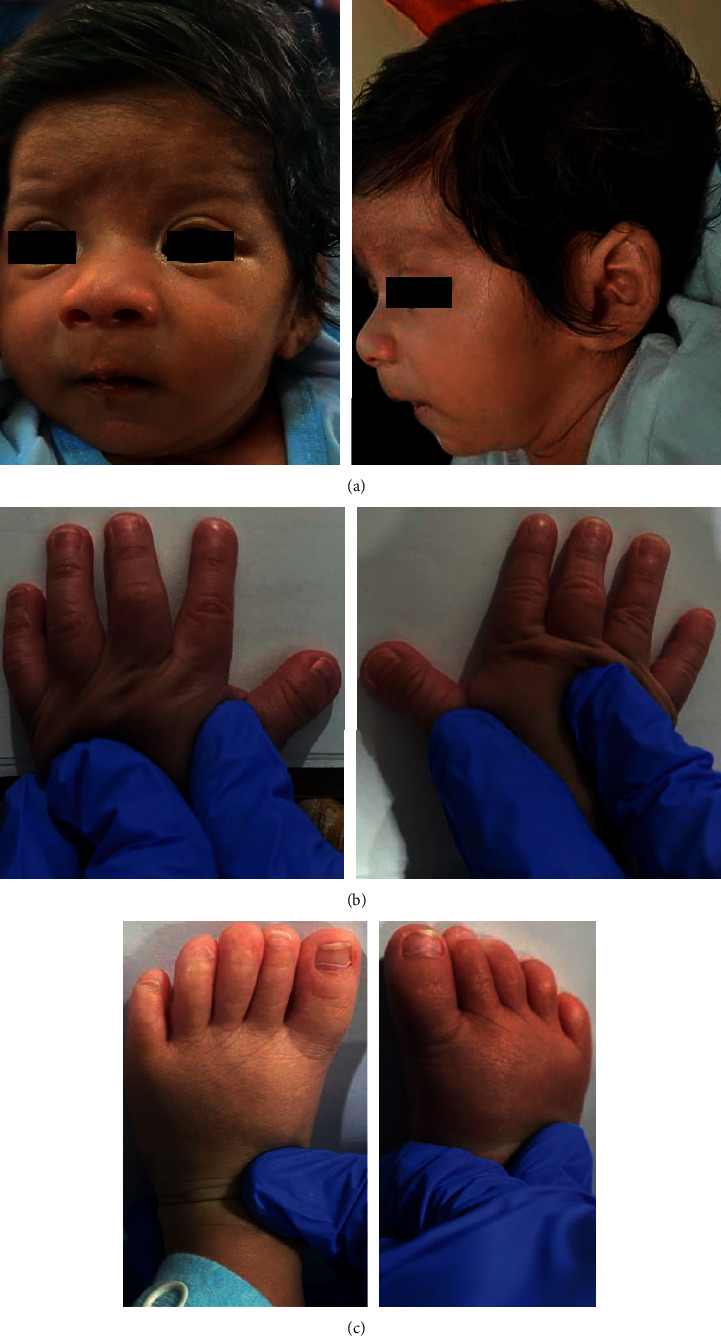
Facial features and hands and feet of the patient at 8 months of age. (a) The patient has low anterior hairline, hirsutism on forehead, smooth philtrum, thin upper lip, and micrognathia. (b) Broad thumbs. (c) Broad first toes (photographs taken with permission).

On physical examination, the patient was appropriate for gestational age with a birth weight of 2720 kg (<25^th^ percentile), length of 47 cm (<25^th^ percentile), and head circumference of 32.5 cm (at 25^th^ percentile). He had low anterior hairline, hirsutism on the forehead, smooth philtrum, thin upper lip, micrognathia, bilateral cryptorchidism, and broad thumbs and halluces (Figure 1).

The patient was referred to the service of genetics wherein a suspicion of Rubinstein–Taybi syndrome and a consideration of a possible fetal alcohol syndrome were made. Further diagnostic investigation was performed. Karyotype analysis showed a normal male karyotype. Genitourinary ultrasonography revealed bilateral undescended testes and bilateral grade I hydronephrosis with normal-sized kidneys. The 2D echocardiography was unremarkable. Hearing screening done at the 1^st^ week and 3^rd^ month of life both showed abnormal results. The patient was eventually discharged, improved, and stable.

In the succeeding months, the child had bouts of recurrent pneumonia which warranted several hospitalizations. This was attributed to a clinically diagnosed gastroesophageal reflux disease for which a gastrostomy feeding tube was inserted as a temporizing measure while awaiting definitive surgical management. On follow-up at 8 months of age, the anthropometric measurements showed severe stunting, wasting, and microcephaly. The previously documented distinct physical features such as a low anterior hairline, hirsutism on the forehead, smooth philtrum, thin upper lip, micrognathia, and broad thumbs and halluces were still apparent. In addition, evaluation of the patient's developmental milestones revealed a mental age of 5 months and the presence of significant delays in all the developmental domains.

Pretest and posttest genetic counselling were done prior to genetic testing, and informed consent was obtained from the patient's parents. Extraction of genomic DNA from the patient's peripheral blood and gene-targeted testing were done at Invitae Corporation (San Francisco, California, USA). The infant was found to have a heterozygous pathogenic deletion at position c.3676 (variant CREBBP NM¬_004380.3 c.3676del p(Ala1226ProfsTer24)) resulting to a frameshift mutation in exon 19 of the *CREBBP* gene. This is predicted to result in a premature translational stop signal, leading to an absent or disrupted protein of the CREBBP gene for which loss-of-function is a known mechanism of disease. The genes of other syndromes with features overlapping with RSTS such as Cornelia de Lange syndrome and floating-harbor syndrome [2] were also tested, but no corresponding pathogenic variants were detected.

## 3. Discussion

This report contributes to the clinical spectrum of RSTS type 1 which is characterized by mutations in the *CREBBP* gene. Described here in detail is the phenotype associated with a novel variant, which according to the ClinVar database has not yet been associated with a specific patient prior to this report. This establishes an important link between a novel variant and its corresponding phenotype. The presence of low anterior hairline, hirsutism on the forehead, micrognathia, microcephaly, bilateral cryptorchidism, broad thumbs and halluces, growth retardation, and developmental delay are all consistent with the diagnosis of RSTS (Table 1). Recurrent respiratory infection is a known complication of RSTS which can be due to gastroesophageal reflux disease (GERD), esophageal anatomic anomalies, or immune deficiency, the most common of which is hypogammaglobulinemia [3–6]. Urologic abnormalities are encountered in approximately 52% of RSTS patients. Cryptorchidism has the highest incidence of about 78% to 100% [3]. The undescended testes seen in the patient may warrant surgical intervention if persistent until 12 months of age. Meanwhile, the postnatal growth retardation and developmental delay are expected in this patient. Growth parameters must be regularly plotted in RSTS-specific growth charts to ensure proper monitoring and management [3, 7]. Prompt recognition and referral to behavioral and developmental specialists are likewise warranted. A neuropsychological assessment should be planned to determine the intellectual quotient (IQ) or the general quotient of development (GQ) of the patient and to highlight the most weakened domains that should be addressed [3].

The cAMP-response-element binding protein (CREB)-binding protein (*CREBBP*) gene encodes for a protein serving as a transcriptional coactivator associated with cell growth and development [8]. The frameshift mutation variant in exon 19 of the *CREBBP* gene of our patient has not been reported in the literature to date. This mutation is pathogenic because it leads to a premature stop codon and is predicted to result in an absent or truncated protein product. This mutation leads to a premature translation stop upstream of the histone acetyltransferase (HAT) domain and thus may have a significant effect in its function in gene expression [9].

The maternal history of heavy prenatal alcohol intake is also striking in this case and predisposes the patient in developing fetal alcohol syndrome (FAS) characterized by facial features of smooth philtrum, thin upper lip and short palpebral fissures, growth retardation, abnormal brain development, and developmental delay [10]. The patient satisfies a majority of these criteria, but since some of the features of RSTS overlap with FAS, the coexistence of the two conditions cannot be fully ascertained. The smooth philtrum is not a characteristic of RSTS but it may be related to the alcohol exposure. It is challenging to give a diagnosis of FAS at this point because the growth retardation and developmental delay can be explained by the *CREBBP* mutation alone. Nevertheless, given the possibility of the coexistence of both syndromes in this patient, more attention must be given in monitoring the patient's growth and neurobehavioral development.

The management of RSTS patients depends on the presenting abnormalities. More than 90% of affected individuals survive into adulthood and most achieve some independence in self-care and communication [3]. Life expectancy is generally normal but may be reduced in RSTS individuals who are particularly susceptible to infections or with severe congenital heart defects; hence, prompt management of these complications is important. They can also present with behavioral disorders, mood swings, and obsessive-compulsive disorders as they grow older until they reach adulthood [3, 4].

The patient's overall clinical presentation was thoroughly explained to the parents and related to the syndrome. Its sporadic nature and the recurrence risk were also discussed with them. They were also provided information about the natural history of the syndrome and directed to important resources and support groups for further guidance. Proper nutrition, adequate follow-up to a developmental specialist for the global developmental delay, and appropriate monitoring and active management of the GERD, cryptorchidism, and hydronephrosis are emphasized. Other essential medical care such as immunizations must be provided as in the general population.

Meanwhile, therapeutic approaches to address the molecular pathology of RSTS are being investigated in various studies. Most are in the preclinical phase of testing. Notable therapeutics under study include histone deacetylase inhibitors (HDACi) which are expected to prevent the epigenetic alterations from CBP/p300 acetyltransferase dysfunction [2]. HDAC inhibitors such as Trichostatin A (TSA) and valproic acid have been used on induced pluripotent stem cell (iPSC)-derived neurons from CREBBP-mutated patients and found to ameliorate the morphological anomalies and electrical activity of young and mature neurons, respectively [11]. Another potential therapeutic target is CBP/CREBBP activation to specifically target CBP/CREBBP-dependent histone acetylation and transcription. The suggested methods include either overexpression of the deficient CBP protein or using a pharmacologic method to activate the CREBBP gene [12]. Given that most genetic mutations are irreversible while epigenetic modifications are highly reversible, therapeutic targeting and modulation of epigenetic components altered in RSTS might be an ideal method and promising future treatment modality.

## 4. Conclusion

RSTS is a rare genetic disease characterized by distinctive physical features, growth retardation, and intellectual disability. Diagnosis is mainly clinical, but the lack of consensus diagnostic criteria to date and the overlap of its features with a myriad of clinical syndromes pose a challenge to its diagnosis. Hence, molecular genetic testing is important for confirmation. Prompt recognition of its manifestations is crucial to ensure timely intervention and prevent debilitating complications. This report contributes to the growing knowledge of RSTS in the Philippines and emphasizes the importance of genetic testing especially in cases wherein the diagnosis can be confounded by the possibility of another coexisting condition or if physical features overlap with other syndromes.

## Figures and Tables

**Table 1:** Patient's clinical features compared to the typical features of RSTS.

Features of RSTS (incidence %) [1]	Patient's clinical features
Typical facial features (100%)	Low anterior hairline, hirsutism, thin upper lip, and micrognathia
Intellectual disability (∼100%)	Global developmental delay
Cryptorchidism (78–100%)	Bilateral cryptorchidism
Microcephaly (35–94%)	Microcephaly
Broad thumbs/halluces (96%)	Broad thumbs and halluces
Speech delay (90%)	Delay in expressive and receptive language domains
Recurrent respiratory infections (75%)	Recurrent pneumonia
Delayed bone age (74%)	—
Constipation (40–74%)	—
Talon cusps (73%)	—
Gastroesophageal reflux (68%)	Gastroesophageal reflux disease
EEG abnormalities (57–66%)	—
Renal anomalies (52%)	Bilateral grade I hydronephrosis
Refractive defects, glaucoma, retinopathy (>50%)	—
Congenital heart defects (24–38%)	—
Seizures (25%)	—
Keloids (24%)	—
Deafness (24%)	2 consecutive abnormal hearing screening results at 1 week and 3 months of age
Growth retardation (21%)	Wasted, severely stunted, and microcephalic at 8 months of age
Malignant tumors (3–10%)	—
Spinal cord tethering (<5%)	—
